# Argon Bioactivation of Implants Installed Simultaneously to Maxillary Sinus Lifting without Graft. An Experimental Study in Rabbits

**DOI:** 10.3390/dj9090105

**Published:** 2021-09-06

**Authors:** Yuki Omori, Daniele Botticelli, Mauro Ferri, Rafael Delgado-Ruiz, Vitor Ferreira Balan, Samuel Porfirio Xavier

**Affiliations:** 1 Department of Oral Implantology, Osaka Dental University, Osaka 565-0871, Japan; info@omori-dent.com; 2 ARDEC Academy, 47923 Rimini, Italy; daniele.botticelli@gmail.com; 3 ARDEC Foundation, Cartagena de Indias 130001, Colombia; medicina2000ctg@hotmail.com; 4 Department of Prosthodontics and Digital Technology, School of Dental Medicine, Stony Brook University, New York, NY 11794-8712, USA; 5 Department of Oral and Maxillofacial Surgery and Periodontology, Faculty of Dentistry of Ribeirão Preto, University of São Paulo, São Paulo 14040-904, Brazil; vitor.balan@usp.br (V.F.B.); spx@forp.usp.br (S.P.X.)

**Keywords:** animal study, sinus floor elevation, bone healing, Schneiderian membrane, histology

## Abstract

Background: The treatment of the surface of titanium implants with argon plasma improved its hydrophilicity and cell adhesion, resulting in higher bone apposition on implant and graft surfaces. The spontaneous perforation over time of the sinus mucosa after sinus augmentation has been documented in experimental studies at both implants and graft particles. The aim of the present study was to evaluate the influence of plasma argon treatment of the implant surface on bone apposition and on the rate of sinus mucosa perforations. Methods: A sinus lifting procedure was performed bilaterally in sixteen rabbits, and implants, either treated with argon plasma or left without treatment (control), were placed simultaneously without grafts. After 8 weeks, histological analyses were carried out. Results: A collapse of the sinus mucosa was observed at all implants. Twenty-four out of thirty-two implants presented sinus mucosa perforations at the apex. Several perforations were also found at the threads. Thinned mucosa sites (width < 40 µm) were found around almost all implants. About 2.6–2.9 mm of the apical regions of the implant did not present signs of osseointegration and about 1.3 mm were exposed to the sinus cavity. No statistically significant differences were found between plasma and control sites. Conclusions: In conclusion, the sinus mucosa was damaged and perforated by direct contact with treated and non-treated implant surfaces. The treatment of the implant surface with argon plasma did not affect the outcomes.

## 1. Introduction

Maxillary sinus floor elevation is often used to increase bone volume in the posterior atrophic region of the maxilla, aiming to allow the placement of implants. The restorations supported by implants in those regions have shown a high success rate [1,2]. However, gaining a high predictability of that treatment is essential for an accurate assessment of a cone beam computed tomography (CBCT), aiming to identify the relevant anatomical parameters [3,4,5,6] and the presence of septa or sinus pathologies [7,8]. The thickness of the sinus mucosa is influenced by several conditions, such as the presence of apical lesions, inaccurate endodontic treatments, severe caries, and periodontal bone loss [9]. The presence of septa might complicate the surgical approach so that an accurate three-dimensional tomographic evaluation is required [10]. It is well known that the subantral space volume created after sinus mucosa elevation will be lost over time [11,12] if no additional measures are taken, such as the placement of biomaterials [13], devices [14,15,16,17] or implants [18,19,20,21,22]. The elevation of the sinus mucosa with simultaneous implant placement and without grafts had been reported as a safe procedure resulting in a high success rate [23]. However, a clinical study showed that the mean bone gain did not reach the apex of all implants meaning that implants were still protruding beyond the new sinus floor without bone support [24]. This was also shown in an experimental study in monkeys [21]. An implant with a moderately rough surface was installed immediately after sinus augmentation without grafts. After one month of healing the sinus mucosa was already found collapsed onto the implant apex and threads. The level of osseointegration from the base of the sinus towards the apex of the implant did not improve between 20 and 30 days of healing, meaning that the collapse of the sinus mucosa hindered new bone apposition toward the implant apex. About 3 mm of the apical region of the implants was protruding inside the sinus without bone. On the other hand, in an experiment in rabbits, implants with a moderately rough surface were placed simultaneously to sinus augmentation with grafts [25]. After 8 weeks of healing, <1 mm of apical region of the implant was not yet integrated into new bone.

Among the complications that might occur during sinus floor elevation, sinus mucosa perforation is the most common [26,27,28,29]. The intentional perforation of the sinus mucosa during standard implant procedures, aiming to make the most of bone crest height and gain a bicortical stabilization, had been described in clinical studies [30,31].

However, if the placement of an implant simultaneously to sinus floor elevation without grafts will results in a protrusion of the implant apex beyond the new sinus floor, the next consequent question is about the fate of the sinus mucosa in such clinical conditions. The spontaneous perforation over time of the sinus mucosa after sinus augmentation has been documented in experimental studies at both implants [32] and graft particles [32,33] so that it might be speculated that implants placed simultaneously to sinus augmentation without graft might be exposed over time to a high rate of sinus mucosa perforations.

Another option to avoid the use of grafts would be that of improving the osteoconductive properties of the implant surface to accelerate bone apposition towards the apex. Several studies have been performed to evaluate the effectiveness of surface modification, applying several different methods [34,35,36,37,38]. Other animal experiments studied the osteoconductivity of different surfaces at marginal defects [39,40,41] showing that the surface modification affects osteoconductivity. It was also shown that a UV treatment of the implant surface improved osseointegration of implants [42]. Additionally, a treatment of titanium implants with argon plasma induces surface modifications, resulting in increased wettability, protein absorption, and cell adhesion [43], as shown by a higher number of adhered cells [44,45,46]. That surface treatment promoted cell differentiation that was attributed to changes in the electric mantel of the titanium surface that attract charged proteins and osteoblasts [47]. It was also shown a stronger fibroblast adhesion to the abutment surface [48], and an increased cell adhesion to various grafts [49]. In animal models, argon plasma treatment increased bone apposition on implants [50] and graft surfaces [51,52,53] in the early phases of healing.

The various properties of the argon plasma treatment illustrated above might protect the sinus mucosa from damages and spontaneous perforations. Moreover, the faster and higher rate of bone apposition might prevent the direct contact of the mucosa with the implant surface. Hence, the present study aimed to evaluate the influence of plasma argon treatment of implant surface on bone apposition and on the frequencies of sinus mucosa damages and perforations.

## 2. Materials and Methods

### 2.1. Ethical Statements

The experimental protocol was approved on 8 April 2019 by the Ethical Committee of the Faculty of Dentistry of Ribeirão Preto, University of São Paulo (protocol No 2019.1.111.58.9). The article was written adhering to the ARRIVE guidelines. The procedures for animal care adopted in Brazil were rigorously followed.

### 2.2. Animal Sample

Sixteen adult male New Zealand white rabbits, weighing approximately 3.5–4.0 kg and 5–6 months old, were used.

### 2.3. Study Design

A randomized split-mouth design was adopted, aiming to eliminate interferences between individuals from the same group. Sinus lifting was performed bilaterally, and implants, one test treated with argon plasma (plasma group), and one without treatment (control group), were placed through the antrostomies without the addition of grafting material.

### 2.4. Sample Calculation

The sample was determined based on preliminary data from a dog experiment in which implants were installed in the edentulous alveolar ridge [50]. A statistically significant difference in bone-to-implant contact in favor for the argon treated implants was obtained with 6 animals. The sample was increased to sixteen animals, considering the different designs exposed to sinus mucosa perforations and the possible loss of implants or animals.

### 2.5. Randomization and Allocation Concealment

The randomization was performed electronically on 26 July 2019 (http://www.randomization.com) by an author not involved in the handling of animals and/or in surgical procedures (SPX). The treatment assignments were kept in opaque sealed envelopes and notified to the surgeon (VFB) immediately after the antrostomies of the maxillary sinuses were completed bilaterally and the sinus mucosa was elevated. The assessor of the histological slides (DB) was blinded about the treatment.

### 2.6. Implants Characteristics and Bioactivation of the Implant Surface with Argon Plasma

**Figure 1:**
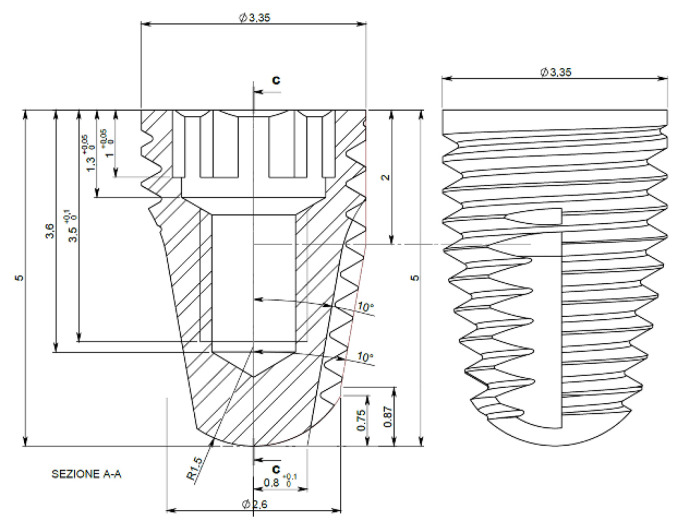
Implant technical dimensions.

The dimensions of the implants were selected based on the outcomes of previous similar experiments performed in rabbits by the same group [25,54]. Thirty-two tapered titanium grade IV implants (Five; Leader Medica, Padua, Italy), 5 mm in length and 3.35 mm in coronal diameter, were used. All implants had the same geometry (Figure 1) and a double acid etched surface. Sixteen test implants underwent a surface treatment with argon plasma. The implants were removed in a sterile mode from the envelope to allow gas and electric charge to get in direct contact with the implant surface. For bio-activation, implants underwent a vacuum cold plasma treatment using a chairside plasma reactor (Plasma R, Sweden & Martina, Padua, Italy). The gas used rate was Ar (>99.9990% purity). After reaching 10-Pa base pressure, Ar was introduced in the reactor chamber. The operating pressure was 20 Pa and the treatment time was 12 min [39].

### 2.7. Surgical Procedures

All surgeries were performed by an expert surgeon (VFB). Acepromazine (1.0 mg/kg; Acepran^®^, Vetnil, Louveira, São Paulo, Brazil) was administrated subcutaneously and xylazine (3.0 mg/Kg; Dopaser^®^, Hertape Calier, Juatuba, Minas Gerais, Brazil) mixed to 50 mg/kg ketamine hydrochloride (Ketamin Agener, União Química Farmacêutica Nacional S/A, Embu-Guaçú, São Paulo, Brazil) was injected IM. Complementary local anesthesia (0.6 mL; Mepivacaine Hydrochloride 2% + 1:100,000 epinephrine Mepiadre 2% + 1:100,000, Nova DFL, Rio de Janeiro, Brazil) was administered.

**Figure 2:**
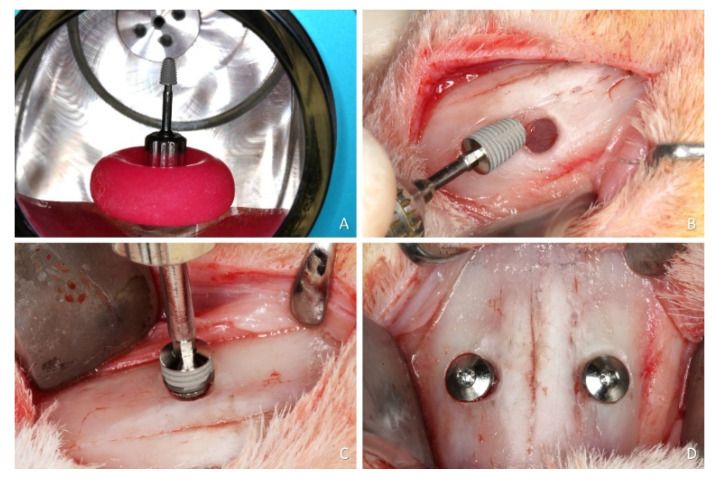
Surgical implantation procedure. (**A**), argon plasma reactor for the treatment of the surface of the test (plasma) implant. (**B**), osteotomy prepared. (**C**), implant placement. (**D**), cover screws were placed on both implants.

After trichotomy and disinfection of the muzzle dorsum, an incision of about 2.5 cm was carried out along the midline. Skin, muscles and periosteum were elevated and the nasal bone was exposed. Round antrostomies, 3.0 mm in diameter, were prepared bilaterally using twist drills. The center of the antrostomies was located about 5 mm lateral to the nasal-incisal suture and 10 mm anteriorly to the nasal-frontal suture. This position corresponds to the sinusal cavities and allows avoiding the nasal cavities, located more mesially. The sinus mucosa was carefully lifted using a small elevator (718-EN1; Bontempi Strumenti Chirurgici, San Giovanni in Marignano, RN, Italy) on the lateral and medial sinus walls as well as mesially and distally to eliminate any tension on the sinus mucosa. Alignment pins were introduced through the antrostomies up to 5 mm of depth from the cortical layer to evaluate the elasticity and integrity of the mucosa. Subsequently, the implants (controls and tests) were inserted through the antrostomies in a random scheme (Figure 2B,C). The primary stability was achieved manually, locking the implant to the nasal bone, paying attention not to break the thin cortical layer.

After the implant placement, cover screws were inserted (Figure 2D), the periosteum was closed with resorbable sutures (Polyglactin 910 5-0, Vicryl^®^, Ethicon, Johnson & Johnson, São José dos Campos, Brazil) and the skin was closed with nylon sutures (Ethilon 4-0^®^, Ethicon, Johnson & Johnson, São José dos Campos, Brazil).

### 2.8. Maintenance Care

The animals were kept in individual cages, accommodated in a climatized room with access to food and water ad libitum. Wounds and biological functions were monitored by veterinarians during the whole period of the experiment. All animals received postoperative medication for 3 days with 1% ketoprofen (Ketofen, Bimeda-Mogivet Farmacêutica SA, 3.0 mg/kg, im; Monte-Mor, São Paulo, Brazil) and tramadol hydrochloride (1 mg/kg, sc, Halexlstar; Goiânia, Goiás, Brazil).

### 2.9. Euthanasia

After 8 weeks, the rabbits were anesthetized with xylazine (3.0 mg/kg IM, Anasedan^®^, Sespo Indústria e Comércio LTDA, Paulínia, São Paulo, Brazil) and ketamine (50.0 mg/kg IM, Dopaser^®^, Sespo Indústria e Comércio LTDA, Paulínia, São Paulo, Brazil). Afterward, the animals were euthanized in a closed transparent acrylic box containing gas carbon dioxide (CO_2_).

### 2.10. Histological Preparation

The nasal region, containing the two filled maxillary sinuses, was harvested in block from all animals and immediately immerged in a 10% formaldehyde buffered solution.

The specimens were dehydrated in a series of alcohols of increasing concentrations and, subsequently, embedded in resin (LR WhiteTM Hard Grid, London Resin Co Ltd., Berkshire, UK) and polymerized. Two sections were obtained from each specimen following the long axis of the implants using precision cutting/grinding equipment (Exakt, Apparatebau, Norderstedt, Germany), and subsequently ground to a thickness of 50–60 µm. The two slides were stained with either toluidine blue or Stevenel’s blue and alizarin red.

### 2.11. Histometric Evaluations

The histological analyses were made by an expert examiner (DB) at both slides using a microscope Eclipse Ci (Nikon Corporation, Tokyo, Japan) connected via a video camera (Digital Sight DS-2Mv, Nikon Corporation, Tokyo, Japan) to a computer. The measurements were made using the software NIS-Elements D (v 5.11.00, Laboratory Imaging, Nikon Corporation, Tokyo, Japan).

**Figure 3:**
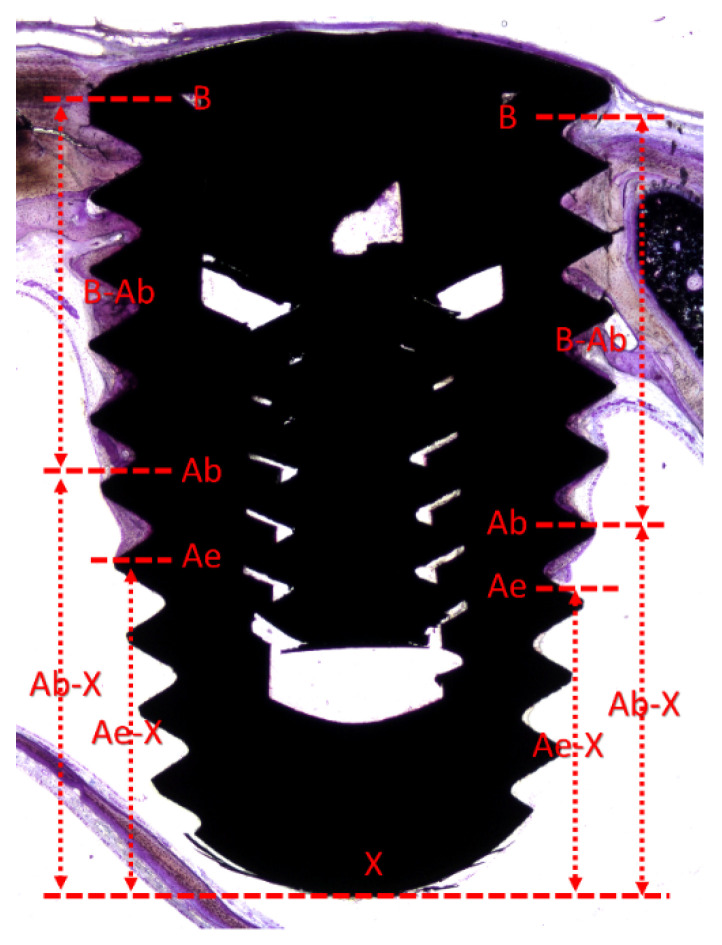
Reference scheme. B, most coronal and most apical (Ab) contacts of the bone to the implant surface; Ae, the apical location of the epithelium of the perforated mucosa; X, apex of the implant. The following vertical measurements were performed: B–Ab, height of osseointegration, Ab–X; apical region of the implant with no osseointegration; Ae–X, apical region of the implant not covered by sinus mucosa.

The following landmarks were used (Figure 3): B, most coronal and most apical (Ab) contacts of the bone to the implant surface; Ae, the apical location of the epithelium of the perforated mucosa; and X, apex of the implant. The following definitions were used: (i) pristine mucosa, the width of the sinus mucosa measured at the lateral and medial sinus walls in regions not involved in the elevation; (ii) apical perforation, interruption of the lining sinus mucosa including the apex; (iii) perforation of the threads, interruption of the sinus mucosa including a thread; thinned mucosa, the regions of the elevated sinus mucosa presenting a width ˂40 µm.

The following assessments were carried out at ×100 magnification: (i) the vertical distances between B and Ab (B–Ab; height of osseointegration), Ab and X (Ab–X; apical region of the implant with no osseointegration), Ae and X (Ae–X; apical region of the implant not covered by sinus mucosa).

The following assessments were carried out at ×400 magnification: (ii) the number of perforations at the apex and at the threads; (iii) the width and number of thinned mucosae sites; (iv) the width of the pristine mucosa.

### 2.12. Data Analysis

The primary variables were Ab–X and Ae–-X, while the secondary variables were the number of perforations and of thinned mucosa sites. The statistical analyses were carried out using Prism 9.1.1 (GraphPad Software, LLC, San Diego, CA, USA). The Wilcoxon test (*p* < 0.05) was applied to disclose differences between the two groups, and the Spearman test (two-tales, *p* < 0.05, 95% confidence interval) was used to evaluate the correlation between Ab-X and Ae-X.

In the tables, mean values, standard deviations, *p* values, medians, and 25% and 75% percentiles were included, while in the text only mean values were reported.

## 3. Results

### 3.1. Descriptive Histological Evaluation

**Figure 4:**
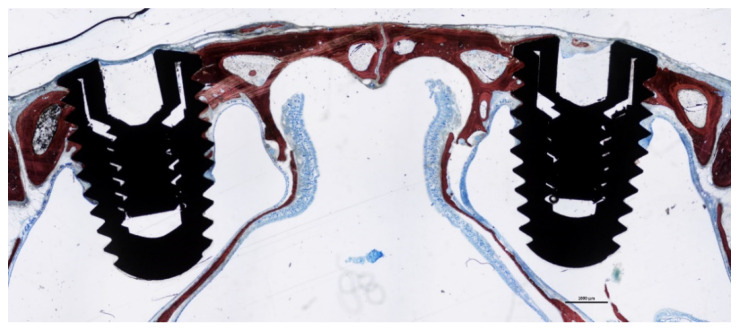
Photomicrographs of a histological ground section showing the position of the implants within the sinus. The primary stability was secured by the cortical layer of the nasal bone.

The positioning of the implants with respect to the sinus and nasal cavities is illustrated in Figure 4.

**Figure 5:**
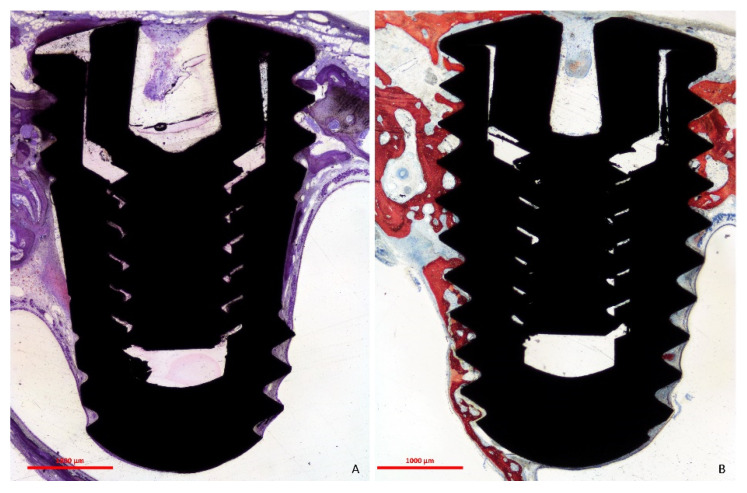
Photomicrographs of ground sections. (**A**), collapse of the sinus mucosa around the implant body with little bone formation from the nasal bone towards the implant apex. (**B**), note bone integration on the implant surface in contact with the medial bone wall of the sinus. (**A**), Toluidine blue stain; (**B**), Stevenel’s blue and Alizarin red.

After 8 weeks of healing, at the histological analysis, the sinus mucosa appeared to be collapsed around the implant body (Figure 5A).

**Figure 6:**
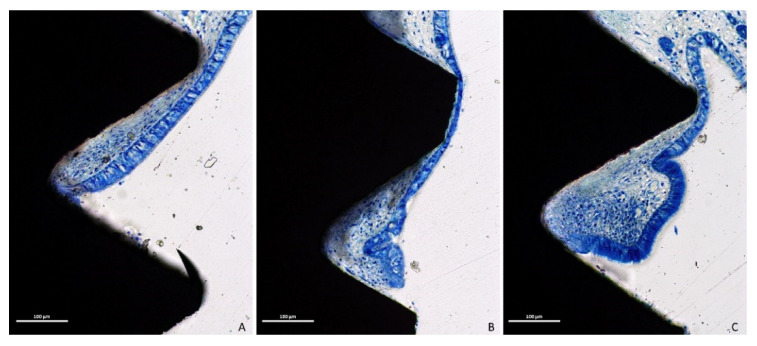
Photomicrographs of ground sections. The perforations were bordered by tapered epithelial cells, presenting in both groups limited (**A,B**) or high (**C**) degrees of inflammatory infiltrate. Stevenel’s blue and Alizarin red stain.

Only eight out of thirty-two implants did not present perforations at the apex. However, two of these eight implants exhibited threads exposed to the sinus cavity so that only six implants were surrounded by mucosa. Moreover, in some cases, the mucosa integrity was lost both at the apex and threads. The perforations were bordered by tapered epithelial cells, presenting in both groups various degrees of inflammatory infiltrates (Figure 6A–C).

The implants devoid of perforations at the apex presented in some instances the apex in contact with the medial bone wall, which contributed to the osseointegration of the medial aspect of the implant (Figure 5B). This might have also contributed to maintaining the integrity of the sinus mucosa.

**Figure 7:**
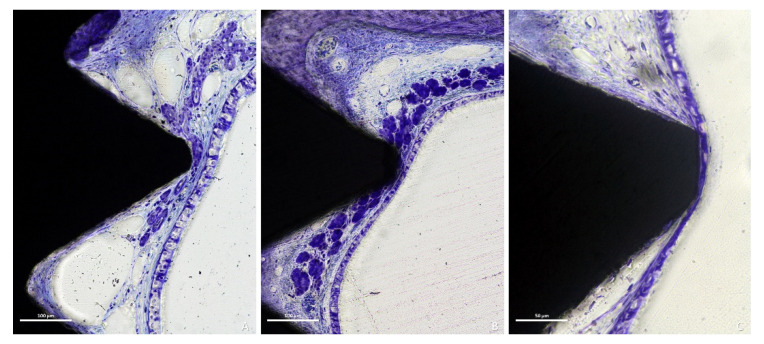
Photomicrographs of ground sections. (**A**), initial stage of thinning mucosa, vessels and mucous glands were dislocated and deformed by the thread tips without damages of the pseudostratified ciliated columnar epithelium. (**B**), more advanced thinning presenting a complete dislocation and deformation of the submucosa, vessels and mucous glands. (**C**), severe stage presenting a progressive thinning of the epithelium layer and a loss of goblet cells and cilia. (**A**–**C**), Toluidine blue stain.

**Figure 8:**
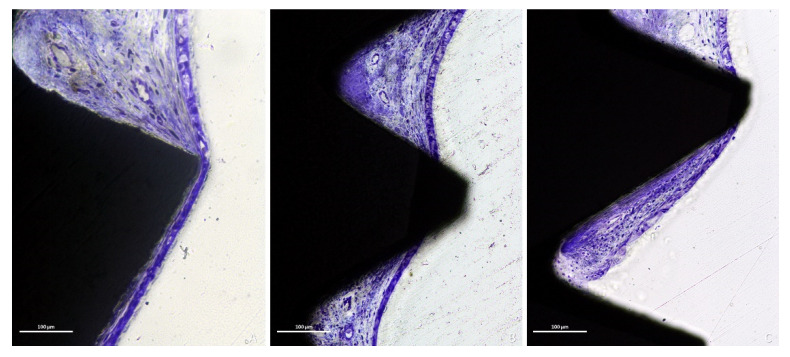
Photomicrographs of ground sections. (**A**), thinned mucosa around the last threads and the apex (at the bottom). (**B**,**C**), the sinus mucosa surrounding the exposed threads generally presented lower amount of inflammatory infiltrated compared to the apical exposure. (**A**–**C**), Toluidine blue stain.

Twenty-seven out of thirty-two implants presented thinned mucosa sites, being <10 µm in thirty-two sites. Only five implants among those severely exposed within the sinus cavity did not present thinned sites. The thinned mucosa sites were mainly associated with the vertex of the implant threads and presented different histological characteristics based on the severity of the damage. In the less compromised sites, vessels and mucous glands were dislocated and deformed by the thread tips without damaging the pseudostratified ciliated columnar epithelium (Figure 7A). However, as the mucosa thickness decreased, a complete dislocation and deformation of the submucosa, vessels and mucous glands (Figure 7B), as well as a progressive thinning of the epithelium layer and a loss of goblet cells and cilia were observed (Figure 7C and Figure 8A).

The sinus mucosa surrounding the exposed threads generally presented a lower amount of inflammatory infiltrated compared to the apical exposure (Figure 8B,C).

**Figure 9:**
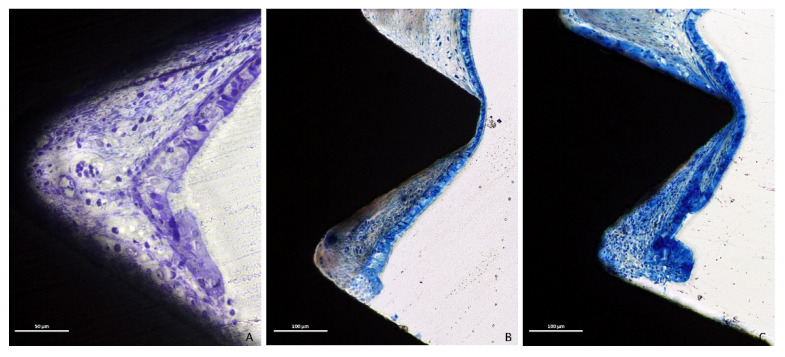
Photomicrographs of ground sections. Repairing tentative to close the exposed submucosa layers was seen performed by the epithelial cells (**A**,**B**), often associated with a hyperfunction of the goblet cells (**A**). The epithelium was also found enfolding coronally underneath the adjacent mucosa (**C**). (**A**), Toluidine blue stain. (**B**,**C**), Stevenel’s blue and Alizarin red stain.

The epithelial cells (Figure 9A–C) were observed close to the exposed submucosa layer. An incomplete reparative process was evidenced associated with the hyperfunction of the goblet cells (Figure 9A). The epithelium was also found enfolding coronally underneath the adjacent mucosa (Figure 9C).

**Figure 10:**
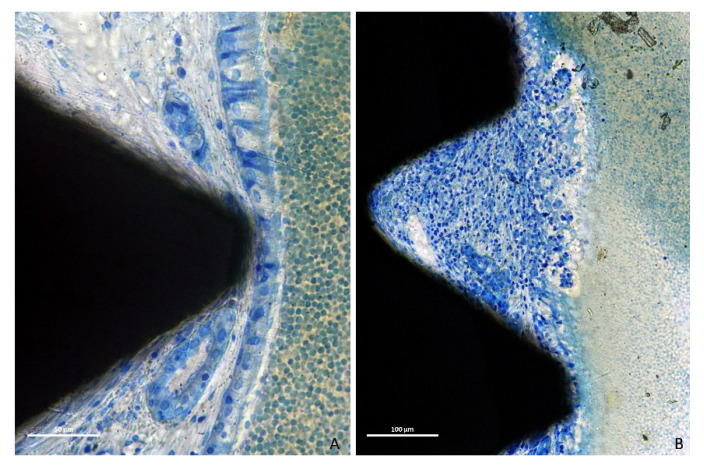
Photomicrographs of ground sections. (**A**,**B**), inflammatory infiltrates within the sinus concomitant to various degree of degeneration in the sinus mucosa were rarely found. (**A**,**B**), Stevenel’s blue and Alizarin red stain.

Inflammatory infiltrates were rarely found within the sinus (sinusitis). They were concomitant to various degree of degeneration the sinus mucosa (Figure 10A,B).

### 3.2. Histometric Evaluation

The mean distance B–Ab (height of osseointegration) was 1.97 mm and 1.81 mm at the plasma and control groups, respectively (Table 1). The mean distances Ab–X and Ae–X were 2.65 mm and 1.32 mm in the plasma group, and 2.90 mm and 1.31 mm in the control groups. None of the differences were statistically significant.

The number of perforations at the apex and threads were similar in both groups, 12 apexes and 15 threads in the plasma group, and 12 apexes and 18 threads in the control group (Table 2). The thinning sites were 55 and 58 at the plasma and control groups, respectively. None of the differences were statistically significant. The correlation coefficient between Ab–X and Ae–X was r = 0.69 (*p* = 0.004: C.I. 0.28–0.89) in the plasma group and was r = 0.67 (*p* = 0.005; C.I. 0.25–0.88) in the control group.

The mean values of the width of the thinning mucosa were 14 µm and 15 µm at the plasma and control groups, respectively (Table 3). The respective mean values of the thinnest sites were 10 µm and 8 µm. The mean pristine mucosa width was 82 µm in the plasma group, and 79 µm in the control group. None of the differences between groups were statistically significant.

## 4. Discussion

The present experiment aimed to evaluate the influence of plasma argon treatment of the implant surface on bone apposition and on the rate of sinus mucosa perforations. The data showed no effect induced by the argon plasma treatment on any of the parameters evaluated.

The risk of contamination, even in a clinical environment, is zero, as demonstrated by an in vitro study [55]. An experimental study did not report any complication of infections at the implant installed in edentulous dog mandibles [50]. In that experiment, the surface of two implants was treated with argon plasma, while two implants were left without treatment. After two months of healing, a statistically significant higher bone-to-implant contact percentage (BIC%) was found in the treated (72.5%) compared to the no-treated implants (64.7%). Nevertheless, when the argon plasma surface treatment was applied to granules of xenograft used for maxillary sinus augmentation in an experiment in rabbits [51], only the furthest regions from the sinus bone walls were positively influenced by that treatment. In another experiment on sinus lifting in rabbits, the treatment with argon plasma of β-TCP/HA granules resulted in a higher bone formation compared to no treated granules when used for sinus lifting in rabbits [52]. However, the difference between the groups was not statistically significant. No effect on healing was finally observed when the argon plasma was used on xenogeneic block grafts secured to the lateral aspects of the mandible angle of rabbits [53].

In the present study, the sinus mucosa collapsed onto the implant body and several perforations occurred, presenting a downward migration of the mucosa from the apical to the coronal region of the implant. This fact might have reduced the chances of the argon plasma treatment to express its potential to promote bone apposition that was instead shown in the previously cited experiment [50]. In fact, in both groups, 1.3 mm of implant length from the apex was not covered by mucosa, a condition that might have reduced the chance for new bone apposition. Consequently, the apical 2.7–2.9 mm of the implant body was not lined by bone after 8 weeks of healing. A positive correlation between Ab–X and Ae–X corroborates this assumption. These outcomes are in agreement with other experimental [21] and clinical studies [24] that showed about 3 mm of protrusion of the implant beyond the new sinus floor. On the other hand, a similar experiment in rabbits in which implants were placed simultaneously to sinus augmentation performed with DBBM granules, < 1 mm of implant apical region was not integrated yet after 8 weeks of healing [25].

Several sinus mucosa perforations at the implant apex and threads were found in both groups. This is in agreement with another experiment in rabbits in which implants were simultaneously placed after bilateral sinus augmentation using either a deproteinized bovine bone mineral (DBBM) or autogenous bone (AB) collected from the tibia [32]. The healing was evaluated after 7 days and 40 days, six animals each period. After 7 days of healing, no perforations were found in the implants, and few contacts between implants and sinus mucosa were observed. After 40 days of healing, three perforations at implants in two sinuses were found at the AB sites and one perforation in one sinus at the DBBM sites. The higher number of perforations at implants at the AB compared to the DBBM sites was ascribed to the higher rate of resorption of the former compared to the latter grafts, an event that exposed the sinus mucosa to a collapse onto the implant body at the AB sites. However, it also has to be considered that the DBBM granules were also associated with perforations of the sinus mucosa. After 7 days of healing, in that experiment [32], while no perforations in the AB particles were found, six perforations in two sinuses were observed in the DBBM granules. After 40 days of healing, three perforations of the mucosa in two sinuses were observed in association with DBBM granules, while no perforations were seen in the AB group, excluding those already described at the implants body.

The perforations of the sinus mucosa in association with grafts were also documented in another experiment in rabbits [33]. Maxillary sinus augmentation was performed bilaterally, and subantral spaces were filled with DBBM composed of either large or small granules. No implants were placed simultaneously. The healing was evaluated after 2, 4 and 8 weeks, six animals each period. Only one perforation was found in one sinus of the large granules group after 2 weeks of healing. The perforations increased progressively in the subsequent periods of healing so that, after 8 weeks of healing, three sinuses out of six in the large granules group, and four sinuses out of six of the small granules group, presented perforations of the sinus mucosa.

In the present study, the pristine mucosa mean width was about 80 µm, similar to that reported in other studies [32,33,56]. In addition, the thinned mucosa (<40 µm) observed around both implant groups was characterized by dislocation and anatomical alterations of the structure, such as glands and vessels, trapped against the prominences of the implant surface. It has been shown that the number of thinned mucosae increased over time both at implants [32] and at DBBM sites [33]. This might be interpreted as the sinus mucosa undergoing a progressive reduction in its width and degeneration, resulting in eventual perforation. In the present study, thinning and perforation of the sinus mucosa were mainly associated with prominences of the implant (apex and threads). Meanwhile, in other studies including graft particles, sinus mucosa thinning and perforation have been correlated with sharp edges and cutting projections of the graft granules [32,33]. After augmentation, the sinus tends to re-pneumatize, resulting in a shrinkage of the subantral volume as documented by an overview [57], systematic reviews [58,59], humans RCTs [60,61,62,63], and several animal experiments [11,12,21,54,64,65]. Perforations were also found in devices used to maintain elevated the sinus mucosa after sinus augmentation procedures in monkeys [14,15,16].

In the clinical practice, the protrusion of the implant above the sinus floor has not been associated with sinus complication. A systematic review [66] reported that the most frequent complications were post-surgical epistaxis and sinus mucosa thickening. It was reported that one single case of sinusitis recovered after antibiotic therapy. Moreover, the height of implant protrusion within the sinus (≤4 mm or >4 mm) did not significantly influence the survival rate and complications. It was concluded that the exposure of dental implants in the sinus cavity without graft materials resulted in a high survival rate (95.6%) after a mean period of evaluation of about 4 years.

Experimental studies in dogs were also performed to assess maxillary sinus complications at implants exposed into the sinus [67,68,69]. After 5–6 months of healing, all implants were integrated, and no pathological events were disclosed in any sinus. The data from the present study are not in complete agreement with those reported above. Even though all implants were integrated, new bone apposition toward the apex was very limited. Moreover, inflammatory infiltrates of various dimensions were found within the soft tissues surrounding the perforations. The epithelium was not always found covering these areas completely so that, in several instances, the soft tissues were exposed to the sinus cavity. In some cases, the epithelium was found wrapped around itself in an attempt to close open wounds. Moreover, even though rarely, signs of localized sinusitis were found.

As a limitation of the present study, it should be considered that the sinus mucosa in rabbits is thinner (~80 µm) than the human sinus mucosa (~0.45–1 mm) [70]. Nevertheless, the thinning of the sinus mucosa observed in the present study and the data reported in other studies described previously allows supposing that similar thinning of the mucosa might occur also in humans, resulting in eventual perforations of the sinus mucosa. A control group at time 0 should have been included to assess a possible surgical trauma to the sinus mucosa, even though in the present study an alignment pin was used before implant installation to verify the elasticity of the sinus mucosa before implant placement. Tomographic and microtomographic examinations could be added to provide 3D information. Shorter and longer periods of healing should be allowed to progressively evaluate the events and to detect possible signs of soft tissue growth in a tentative of reparation.

From a clinical point of view, the outcomes from the present study suggest that a perforation of the sinus mucosa might occur at implants protruding beyond the new sinus floor. Nevertheless, the success rate of these implants is high and the frequency of complications is low [66].

## 5. Conclusions

In conclusion, the sinus mucosa was damaged and perforated by direct contact with treated and non-treated implant surfaces. The treatment of the implant surface with argon plasma did not affect the outcomes.

## Figures and Tables

**Table 1:** Histometric measurements in millimeters of hard and soft tissues on the implant surface.

		B-Ab	Ab-X	Ae-X
Plasma	Mean ± SD Median (25%; 75%)	1.97 ± 0.91 1.84 (1.22; 2.61)	2.65 ± 0.94 2.72 (1.98; 3.39)	1.32 ± 1.31 0.84 (0.08; 2.39)
Control	Mean ± SD Median (25%; 75%)	1.81 ± 0.82 1.67 (1.17; 2.23)	2.90 ± 0.92 3.12 (2.38; 3.64)	1.31 ± 1.36 0.86 (0.09; 2.32)
	Wilcoxon test	0.678	0.520	0.891

B, most coronal and most apical (Ab) contacts of the bone to the implant surface; Ab, most apical contact of the sinus mucosa epithelium to the implant surface; X, apex of the implant. most apical (Ab) contacts of the bone to the implant surface. *p* ˂ 0.05.

**Table 2:** Number of perforations and thinning mucosa sites.

	Perforations at Apex	Perforations at Threads	Thinning Sites
Plasma	12	15	55
Control	12	18	58
Wilcoxon test	1.000	1.000	0.826

*p* ˂ 0.05.

**Table 3:** Histometric measurements in micromillimeters of the mean values of the thinning, lowest and pristine mucosa widths.

		Thinning Mucosa Width	Thinnest Mucosa Width	Pristine Mucosa
Plasma	Mean ± SD Median (25%; 75%)	14 ± 9 15 (9; 21)	10 ± 9 7 (2; 19)	82 ± 15 83 (68; 93)
Control	Mean ± SD Median (25%; 75%)	15 ± 8 16 (9; 21)	8 ± 7 7 (3; 11)	79 ± 15 77 (74; 87)
	Wilcoxon test	0.592	0.608	0.440

*p* ˂ 0.05.

## Data Availability

The data are available on reasonable request.
